# Modulation of Production of Th1/Th2 Cytokines in Peripheral Blood Mononuclear Cells and Neutrophils by Hepatitis C Virus Infection in Chronically Infected Patients

**DOI:** 10.3390/pathogens10111519

**Published:** 2021-11-21

**Authors:** Sahar Essa, Iqbal Siddique, Motaz Saad, Raj Raghupathy

**Affiliations:** 1 Department of Microbiology, Faculty of Medicine, Kuwait University, Kuwait City 24923, Kuwait; raj.raghupathy@ku.edu.kw; 2 Department of Medicine, Faculty of Medicine, Kuwait University, Kuwait City 24923, Kuwait; iqbal.siddique@ku.edu.kw; 3 Thunayan Al-Ghanim Gastroenterology Center, Al-Amiri Hospital, Kuwait City 24923, Kuwait; 4 Hepatology and Gastroenterology Unit, Mubarak Al-Kabir Hospital, Jabriya 46300, Kuwait; motaz_mowafy@yahoo.com

**Keywords:** hepatitis C virus, HCV genotypes, neutrophils, Th1, Th2, liver disease progression

## Abstract

This study investigated the influence of Hepatitis C virus (HCV) infection on the cytokine production profiles of the peripheral blood monoculear cells (PBMC) and neutrophils in chronically naïve HCV-infected patients. Seventy-five genotype-4 naïve HCV-infected patients (HCV+) and healthy subjects (HCV−) were enrolled. The neutrophils and the PBMC were separated by density gradient sedimentation and stimulated with a mitogen. The culture supernatants were evaluated for levels of IFN-α, IFN-γ, IL-2, IL-4, IL-5, IL-6, IL-9, IL-10, IL-12, and TNF-α using anti-cytokine antibody MACSPlex capture beads. The PBMC cytokine profiles of HCV+ patients showed significantly lower mean values for IFN-γ, IL-2, IL-6, IL-9, and IL-10 (*p* < 0.0001) as compared to HCV− subjects. In contrast, HCV+ patients showed higher mean levels of PBMC cytokine values for IL-5 and TNF-α (*p* < 0.0001). As for neutrophils, HCV+ patients showed significantly lower mean levels of IFN-α, IFN-γ, IL-2, IL-4, IL-6, IL-9, and IL-10 (*p* < 0.0001). In contrast, the neutrophils from HCV+ patients showed higher mean levels of IL-5, IL-12, and TNF-α (*p* < 0.0001). Th1–Th2 cytokine ratios suggested a lower Th1 bias in HCV+ subjects as compared to HCV− subjects. Our results suggest that chronic HCV infection brings about an immunomodulatory effect not only on neutrophils, but also to a lower extent on PBMCs

## 1. Introduction

Neutrophils are basic effector components in inflammatory processes. They are among the first cells to reach the site of infection and have a wide range of effector mechanisms, including phagocytosis, production of oxygen radicals, secretion of cytokines and chemokines, formation of neutrophil extracellular traps, and interactions with other immune cell populations [1,2,3]. Neutrophils, when activated by invading pathogens, secrete multiple pro- and anti-inflammatory cytokines, including interferon (IFN), interleukin (IL)-1, IL-6, IL-8, IL-10, IL-17, and tumor necrosis factor (TNF) [2,3,4,5]. Innate immunity is thought to play an important role in controlling hepatitis C virus (HCV) infection, although the exact mechanisms are not well defined [6]. The induction of proliferation and cytokine production by lymphocytes using mitogens (e.g., phytohemagglutinin, concanavalin A, etc.) has enabled the characterization of Th1 and Th2 responses [7]. Several lines of research have suggested that a preferential shift towards either Th1 or Th2 reactivity in the host affects the clinical outcomes and the progression of diseases [8,9,10,11]. The profiles of two Th1 cytokines and two Th2 cytokines in HCV patients with different clinical outcomes were studied previously [8]. A preferential shift towards a Th1 profile was reported to be associated with a more favorable clinical outcome.

In contrast, a Th2 profile appears to be implicated in HCV pathogenesis and disease severity [12]. Strong evidence exists to show that a specific, robust, and sustained Th1 cytokine response contributes to HCV infections progressing to resolution [13,14]. On the other hand, chronic HCV infection has been associated with a weak Th1 cytokine response with limited specificities and low levels of Th1 cytokines [8,9,10,11].

Interestingly, the role of neutrophils in anti-viral immunity is not yet fully understood [2,3,15]. While neutrophils are the first and major immune cell population recruited to the site of viral infection [2,3], the extent of their contribution and relative importance in antiviral immunity is still unclear. Functionally, neutrophils have been suggested to play a defensive role [2,3], as depletion of these immune cells leads to higher viral loads and subsequent death in a neurotropic JHM strain of mouse hepatitis viral infection [16] and an increase in viral multiplication and fatality in herpes simplex virus type-1 infection of the murine cornea [17]. In addition, neutrophils have been shown to contribute to the control of viral replication and the severity of neurological diseases [18]. Studies have shown intriguing cytokine effects on neutrophils: IFN-γ and TNF-α induce phagocytosis [19] and the intracellular killing of microorganisms [20].

While some studies have documented the immunomodulatory effect of HCV infection in inflammation and antiviral immune responses [3,21,22,23], the impact of HCV infection on the cytokine production patterns of neutrophils has not been well characterized. In the present study, we investigated the effects of HCV infection on cytokine production profiles of the peripheral blood mononuclear cells (PBMC) and the neutrophils in naïve, chronically HCV-infected patients with compensated liver disease. By studying the cytokine profiles of the PBMC and the neutrophils in HCV-infected patients, we hoped to ascertain whether HCV infection brings about any immunomodulatory effects on the neutrophils in chronically HCV-infected patients. Findings from this study will add to our understanding of the immunomodulatory effects of HCV on host immune responses during chronic HCV infection.

## 2. Results

### 2.1. Subjects

We initially recruited 86 chronically naïve HCV-infected patients with compensated liver disease, as well as 75 healthy controls (age- and sex-matched). Patients were inducted from two hospitals in Kuwait (Mubarak Al-Kabeer and Al-Amiri). The 86 subjects were genotyped; 75 of them were genotype-4, and these subjects were included in this study. Table 1 provides demographic and clinical information of the recruited subjects.

### 2.2. Comparison of Levels of Cytokines Produced by PBMC and Neutrophils from HCV+ Patients and Healthy Subjects

**Figure 1:**
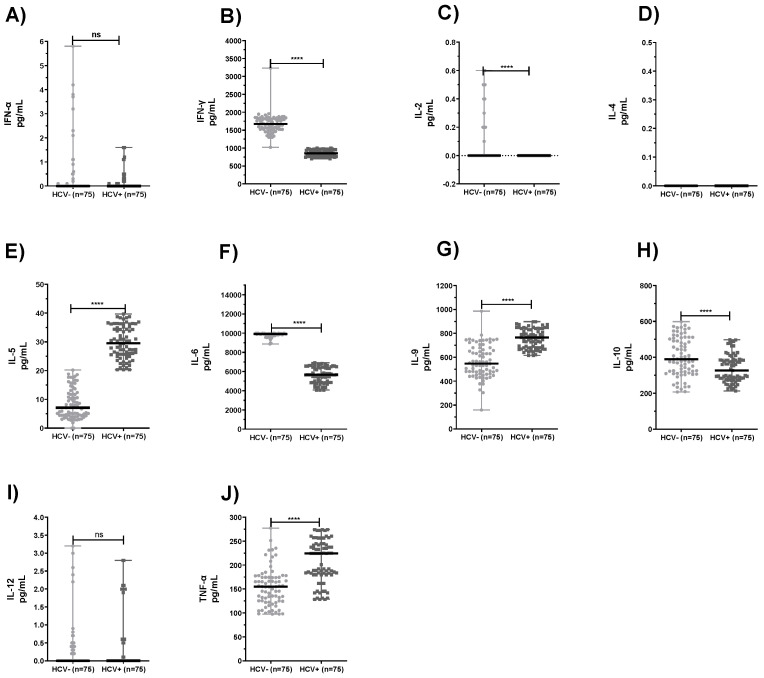
Cytokine profiles of Peripheral Blood Mononuclear Cells (PBMC) from healthy subjects (HCV−) and HCV-infected patients (HCV+). **** *p* < 0.0001 refers to the statistical differences. (ns) refers to no statistically significant differences. (**A**) IFN-α, (**B**) IFN-γ, (**C**) IL-2, (**D**) IL-4, (**E**) IL-5, (**F**) IL-6, (**G**) IL-9, (**H**) IL-10, (**I**) IL-12, and (**J**) TNF-α.

Figure 1 illustrates the statistical comparison of the PBMC cytokine profiles between the HCV+ and HCV− subjects. Significantly lower mean values were observed in the HCV+ PBMC production of IFN-γ, IL-2, IL-6, and IL-10 (Figure 1B,C,F,H; *p* < 0.0001). In contrast, statistically higher mean levels of IL-5, IL-9, and TNF-α (Figure 1E,G,J; *p* < 0.0001) were produced by the PBMC in HCV+ patients. However, no statistically significant differences were detected in the production of IFN-α, IL-4, and IL-12 when the HCV-infected patients were compared with the healthy subjects (Figure 1A,D,I; *p* > 0.05).

**Figure 2:**
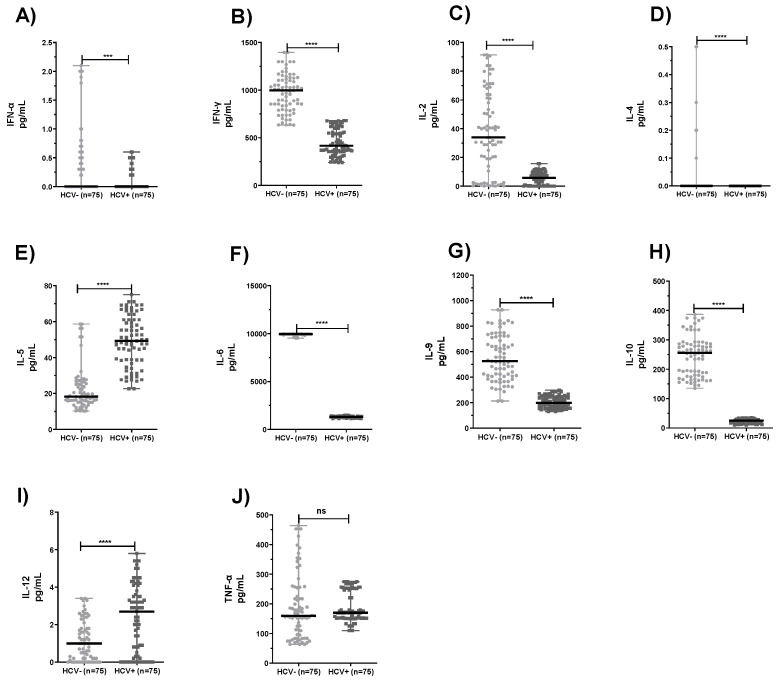
Cytokine values of neutrophils from healthy subjects (HCV−) and HCV-infected patients (HCV+). *** *p* < 0.001, **** *p* < 0.0001 refers to the statistical differences. (ns) refers to no statistically significant differences. (**A**) IFN-α, (**B**) IFN-γ, (**C**) IL-2, (**D**) IL-4, (**E**) IL-5, (**F**) IL-6, (**G**) IL-9, (**H**) IL-10, (**I**) IL-12, and (**J**) TNF-α.

The cytokine production profiles of neutrophils from HCV+ patients and HCV− subjects are shown in Figure 2. Significantly lower mean values were seen in the levels of IFN-α, IFN-γ, IL-2, IL-4, IL-6, IL-9, and IL-10 (Figure 2A–D, F–H; *p* < 0.0001). In contrast, we observed higher mean levels of IL-5 and IL-12 (Figure 2E, I; *p* < 0.0001). However, no significant differences were detected in the levels of TNF-α when the HCV-infected patients were compared to the healthy subjects (Figure 2J; *p* > 0.05).

**Figure 3:**
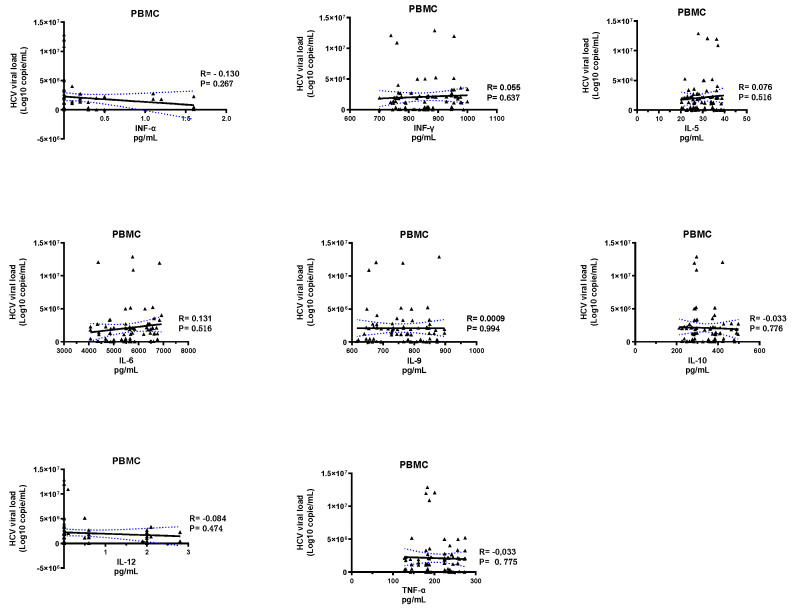
Correlations between cytokine profiles by PBMC and HCV viral load.

**Figure 4:**
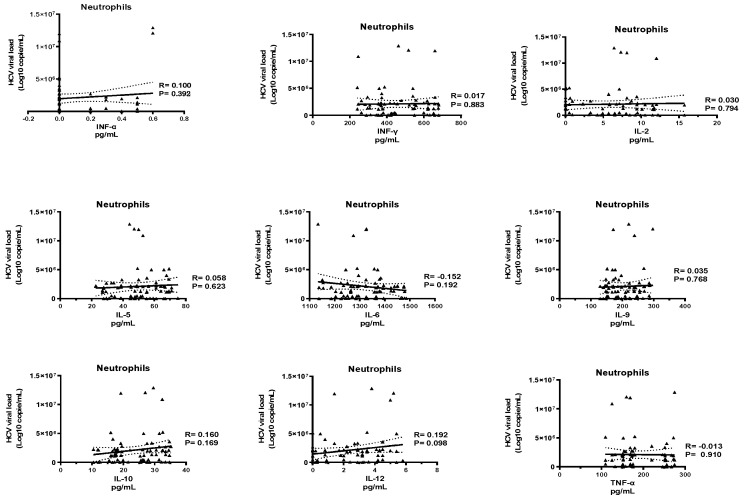
Correlations between cytokine profiles by neutrophils and HCV viral load.

Our results showed no correlation between the ten cytokines tested and the HCV viral load (Figure 3 and Figure 4).

### 2.3. Th1–Th2 Cytokine Ratios

Keeping in mind that the relative levels of the Th1 and Th2 cytokines are as important as, if not more important than, the absolute levels of cytokines, per se, we calculated the ratios of Th1–Th2 cytokines, as these ratios can indicate the dominance of the cytokine patterns. As shown in Table 2, a lower Th1-cytokine bias was seen in the PBMC of the HCV+ subjects in 17 out of the 24 possible combinations. The ratios of IFN-α/IL-5, IFN-α/IL-9, IFN-α/IL-10, IL-2/IL-5, IL-2/IL-9, and IL-2/IL-10 were substantially lower (43,333-, 19,000-, 1000-,767-, 300-, and 167-fold differences, respectively) in the HCV+ patients than the HCV−. Only in 5 out of the 24 combinations were higher Th1-cytokine biases seen in HCV+ individuals. The lower Th1-cytokine bias in 17 out of the 24 combinations, when compared to a higher Th1-cytokine bias in 5 out of the 24 combinations in the PBMC cytokine profiles, suggested decreased Th1 immunity in the HCV+ subjects. As for the cytokine production profiles of the neutrophils, a lower Th1-cytokine bias was seen in 12 out of the 24 possible combinations in the HCV+ individuals, as compared to the HCV− individuals (Table 2).

The ratios of IL-6/IL-5, IL-6/IL-4, IFN-γ/IL-5, and IFN-α/IL-5 showed lower differences in the HCV+ patients versus the HCV− individuals (143-, 75-, 20.4-, and 20-fold, respectively). In contrast, a stronger Th1-cytokine bias was seen in 12 out of the 24 combinations in the neutrophils of the HCV+ patients, as compared to the HCV− individuals’ ratios involving IL-2, which showed higher differences in the HCV+ versus HCV− subjects. The ratios of IL-2/IL-9 and IL-2/IL-10 were 10,000- and 1000-fold, respectively. In addition, IL-2/IL-4 and IL-2/IL-5 ratios were 58- and 30-fold higher, respectively, in the HCV+ patients.

## 3. Discussion

Neutrophils play a vital role in innate immunity [1,2]. We hypothesized that an HCV infection could modify neutrophil responses and that this would be reflected in their cytokine production patterns. This working hypothesis was supported by the fact that chronic HCV infection modulates antiviral and cytokine immune responses [3,21,22,23]. The results of the present study demonstrated a significant decrease in the production of the three cytokines, IFN-γ, IL-2, and IL-6, by the PBMC and the five cytokines, IFN-α, IFN-γ, IL-2, IL-6, and IL-9, by the neutrophils. In contrast, a significant increase was detected in the three cytokines, IL-5, IL-9, and TNF-α, by the PBMC and the two cytokines, IL-5 and IL-12, by the neutrophils.

IFN-α has been used for the treatment of chronic HCV infections [24]. Pegylated IFN-α combined with ribavirin led to sustained viral clearance in 50% of patients [25]. Thus, any immunomodulatory effect by HCV on IFN-α production may directly affect the outcome of an HCV infection and could exacerbate the infection. Conversely, IFN-γ has been shown to extend neutrophil recruitment to the site of infection [26]. Specific CD4+ T cells regulate the adaptive response by secreting the Th1 cytokines IL-2 and IFN-γ to facilitate a cell-mediated immune response [27]. In addition, IFN-γ induces phagocytosis in neutrophils [19] and the intracellular killing of microorganisms [20]. Thus, the immunomodulation of the neutrophils’ IFN-γ production may prevent the elimination of the virus, leading to chronic hepatitis [28]. Sasaki et al., detected significantly higher IFN and Th1/Th2-related cytokine levels in rapid virological responders treated with direct-acting antivirals, as compared to those of end-of-treatment responders [29].

IL-2 exerts a wide range of remarkable effects on the immune system [30]. In chronic hepatitis C, a decrease in IL-2 appears to be chiefly responsible for the lack of activation of virus-specific CD4+ T cells [13]. Semmo et al. demonstrated that the loss of IL-2 production during chronic HCV infection might interfere with the proliferative function of IFN-γ in both CD4+ and CD8+ T cells [31].

IL-6 is a potent cytokine that controls a wide range of in vivo activities and is recognized for its role in shifting from nonspecific innate immunity to extremely specific, adaptive immunity against infection [32,33]. Furthermore, IL-6 regulates T-cell growth, differentiation, and inhibition of lymphocyte apoptosis to guarantee an effective adaptive immune response [34]. IL-6 is produced by various immune cells and influences the migration, the polarity, and the speed of neutrophils [35,36]. In this study, the significantly lower levels of IL-6 produced by both PBMC and neutrophils may indicate an immunomodulatory effect of HCV, which may reduce the protective role of IL-6 in rescuing lymphocytes from apoptosis and functional exhaustion in chronic HCV infection. A study by Naseem et al. supported the positive role of IL-6 in rescuing the PBMC population and recommended its usage in combination therapy for chronic HCV infection [34].

Our results showed a significant increase in the production of IL-9 by the PBMC in HCV-infected patients as well as a significant decrease in the neutrophils. A similar result in the PBMC in HCV-infected patients was documented by Ali et al., suggesting a direct link between the production of IL-9 and liver disease progression [37]. Similarly, another study indicated that increased levels of IL-6 and IL-9 were prognostic for the failure of HCV therapy, whereas lower levels of IL-9 were associated with the resolution of HCV [38]. Furthermore, the production of IL-5, a Th2 cytokine responsible for regulating humoral immune responses [39], showed a significant increase in PBMC and neutrophils. Similarly, serum levels of IL-5 were reported to be increased in chronically infected HCV patients, as compared to normal controls [40].

We observed a significant increase in the production of TNF-α by the PBMC in HCV+ patients. Previous studies have reported a similar increase in TNF-α levels by the resultant PBMC, thyrocytes, and monocytes of HCV infection [41,42,43]. As for IL-12, a previous study shed light on the importance of IL-12 on the stimulation of PBMC in HCV-infected patients when co-cultured with HCV antigens [44]. A significant increase in the proliferation of PBMC was observed in the presence of IL-12. Our results showed a significant increase in the production of IL-12 by the neutrophils in HCV+ patients. Similar results were documented in monocyte-derived macrophage subsets (i.e., M2a–c), which exhibited increased IL-12 production [45]. The higher levels of IL-12 secreted by neutrophils may help in the stimulation of the PBMC proliferation.

Previous studies investigated the Th1/Th2 bias during HCV infection [3,12,40,46,47], but this has not been studied in terms of cytokine production by neutrophils. We observed a lower Th1-cytokine bias, both in the PBMC and the neutrophils, in HCV+ subjects. Th1–Th2 cytokine ratios revealed an interesting insight. A lower inflammatory cytokine bias was seen in the majority (i.e., 17 out of 24) of the possible combinations for the PBMC in HCV+ subjects. In addition, the Th1–Th2 cytokine ratios in neutrophils showed a lower inflammatory cytokine bias in 12 out of the 24 combinations. The stronger Th1-biased reactivity pattern in neutrophils in HCV+ patients in 12 combinations indicated that neutrophils might compensate, at least to some extent, for the low pro-inflammatory cytokine bias in the 17 out of 24 combinations of the PBMC. These data suggested an immunomodulation of the cytokine responses for the PBMC and the neutrophils induced by chronic HCV infection.

## 4. Materials and Methods

### 4.1. Subjects

A statistical power analysis was conducted to determine the sample size to be studied; the sample size obtained by power calculation for the simple random sample was 75 subjects, assuming a 95% rate of change in HCV infection, a 95% confidence interval, and a maximum accepted error of 0.05. Eighty-six naïve adult chronic HCV patients with compensated liver disease (HCV+) (Child–Pugh–Turcotte class A), aged 18 years old and above, were selected from the Al-Amiri Hospital and the Mubarak Al-Kabeer Hospital in Kuwait. The same number of healthy control subjects (HCV−) were recruited. Criteria for inclusion in the study were the presence of anti-HCV antibody, HCV RNA detectable by a polymerase chain reaction, fluctuating or persistently elevated alanine aminotransferase (ALT) and aspartate aminotransferase (AST) (>1.5-fold of normal levels), and compensated liver disease (Child–Pugh–Turcotte class A). Criteria for exclusion included clinical evidence of hepatic decompensation (e.g., presence of ascites, hepatic encephalopathy, variceal hemorrhage), serum albumin <30 g/L, total serum bilirubin >50 μmol/L, prothrombin time >4 s above control, serum creatinine >140 mmol/L, hemoglobin <110 g/L, total leukocyte count < 2 × 10^9^/L, eosinophil count > 350/μL, platelets < 50 × 10^9^/L, seropositivity for hepatitis B virus (HBV) and human immunodeficiency virus (HIV), alcohol or drug dependence, serious comorbid conditions, severe psychiatric disorders, treatment with antiviral or immunosuppressive agents before enrollment, and diabetes.

This three-year study was initiated after obtaining ethical approval from the Human Research Committee of the Ministry of Health from February 2018 to January 2021. Written informed consent was obtained from all participants. Eighty-six sex- and age-matched healthy control subjects were included. Healthy control subjects were screened for HBV, HCV, and HIV, and only candidates negative for these tests were included in the study. Furthermore, healthy control subjects were tested for ESR and CRP, and only candidates negative for these tests were included in the study to exclude active inflammatory disorder. Eight milliliters of venous blood was collected in EDTA tubes from each subject before antiviral treatment. A fresh sample was obtained from each patient and processed within one hour for PBMC and neutrophil isolation. Additional samples were subjected to the following laboratory investigations: complete blood count (using CELL-DYN 1700); liver function tests including ALT, AST, total bilirubin, direct bilirubin, and serum albumin (using HITACHI 912 automatic analyzer, Roche Diagnostics, Mannheim, Germany) as well as α-fetoprotein (VIDAS autoanalyzer, BioMérieux, France); and, finally, viral load was measured by Quantitative HCV RT-PCR (Artus HCV RG RT-PCR Kit, QIAGEN, Helden, Germany) and HCV diagnostic tests as well as HCV genotyping (INNO-LiPA 2.0, Innogenetics Inc., Ghent, Belgium).

### 4.2. Isolation of PBMC

The PBMC were isolated from fresh whole blood by Ficoll-Hypaque density-gradient centrifugation (Pharmacia Biotech, Uppsala, Sweden) as described previously in [48]. Freshly isolated PBMC were suspended in RPMI medium (Gibco BRL, New York, NY, USA) supplemented with 10% fetal bovine serum (FBS) (Eurobio).

### 4.3. Isolation of Neutrophils

Neutrophils were isolated from fresh whole blood using a mixture of sodium metrizoate and Dextran 500 [49]. Briefly, whole blood was layered over density-gradient medium and centrifuged, the neutrophil layer was isolated, and residual erythrocytes were lysed. Neutrophils were then washed, counted, suspended in the buffer at a cell density of 10^5^ cells/mL, and stimulated as described previously in [48]. The purity and viability were assessed by labeling the cells with neutrophil-specific marker CD66b and trypan blue dye exclusion. Previous publications documented the use of PHA for neutrophil stimulation [50,51].

### 4.4. Mitogen Stimulation

Neutrophil and PBMC were aliquoted separately into 96-well tissue culture plates at a density of 10^5^ cells per well and then stimulated with PHA (Sigma-Aldrich, St. Louis, MO, USA) at a concentration of 5 μg/mL for 96 h. The proliferation of PBMC and neutrophils by PHA was evaluated by radiolabeled thymidine uptake. Our standardization experiments have shown that optimal proliferation of PBMC and neutrophils was seen at 96 h (data not shown). Each sample was cultured with PHA alone to serve as a control. Supernatants were collected for cytokine evaluation on day four and stored at −80 °C.

### 4.5. Evaluation of Cytokines

Supernatants from cultures of PBMC and neutrophil were tested for cytokine production by using the flow cytometry bead-based array with a MACSPlex Cytokine kit, according to the manufacturer’s instructions (Miltenyl Biotec, Bergisch Gladbach, Germany). MACSPlex Cytokine kit detects human IFN-α, IFN-γ, IL-2, IL-4, IL-5, IL-6, IL-9, IL-10, IL-12, and TNF-α. Samples were developed using a MACSQuant Analyzer 10 and were evaluated using the Express Mode option of the MACSQuantify 2.8 software (Miltenyi Biotec, Bergisch Gladbach, Germany).

### 4.6. Statistical Analysis

Statistical significance was analyzed using Prism 9 software (GraphPad Software Inc., San Diego, CA, USA). Viral loads were converted on a logarithmic scale (log10) for statistical analysis. The correlations between cytokines and HCV viral loads were established using Pearson’s correlation coefficient. All statistical analyses were two-sided, and *p* < 0.05 was considered statistically significant and designated as * *p* < 0.05, ** *p* < 0.01, and *** *p* < 0.001. Standard deviation (SD) was represented by error bars.

## 5. Conclusions

Our results shed light on the immunomodulatory effect of HCV infection on both PBMC and neutrophils in naïve, chronically HCV-infected patients. This study provides new information on the effects of HCV infection on neutrophils. A well-accepted dogma in immunology is that effective host immunity in chronic HCV infection involves producing a robust Th1-type (i.e., pro-inflammatory) response and lower Th2-type cytokine response. The results described above suggest that chronic HCV infection activates an immunomodulatory effect, not just on the PBMC (which had been reported in earlier studies), but also on the neutrophils, which had not been described previously in the literature. A lower Th1-cytokine bias was detected in the Th1–Th2 cytokine ratios for both the PBMC and the neutrophils. Due to the complex nature of the cytokine networks, the exact roles of these cytokines in HCV infection/disease are not yet completely understood. Additional studies will be required to examine these mechanisms, which will be valuable for designing effective antiviral therapies. The limitations of this study were the small sample size, 95% purity of neutrophils isolation assay, and that the bulk of participants were genotype-4; the availability of other genotypes would have allowed enhanced comparative results. Studies on larger sample sizes and better purity neutrophil isolation techniques would offer valuable information that would strengthen the conclusions of this study.

## Figures and Tables

**Table 1:** Demographic and clinical data of recruited healthy subjects (HCV−) and naïve HCV-infected patients (HCV+).

Group	HCV−	HCV+
No.	75	86
Gender (M/F)	39/36	45/40
Age (mean ± SD) [range], years	32 ± 10.35 (32–55)	39 ± 9.21 (32–55)
Viral load mean ± SD [range], IU/mL	ND	2.1 × 10^6^ ± 2.7 × 10^6^ (325–11,948,083)
HCV genotype (1/3/4)	ND	4/7/75
AST (mean ± SD, IU/mL)	ND	64.58 ± 33.45
ALT (mean ± SD, IU/mL)	ND	82.78 ± 55.45
ALP mean ± SD, IU/mL	ND	152.65 ± 39.77

Data are shown as mean; ND = Not determined.

**Table 2:** Ratios of pro-inflammatory to anti-inflammatory cytokines in HCV− and HCV+ subjects.

Th1/Th2	PBMC		Neutrophils	
	HCV−	HCV+	HCV−	HCV+
IL-2/IL-4	13	100	10	580
IL-2/IL-5	2.3	0.003	0.004	0.12
IL-2/IL-9	0.03	0.0001	0.000003	0.03
IL-2/IL-10	0.05	0.0003	0.0002	0.2
TNF/IL-4	300	224,600	85,500	16,980
TNF /IL-5	53.8	7.6	32.9	3.4
TNF/IL-9	0.8	0.3	0.2	0.9
TNF/IL-10	1.1	0.7	1.9	6.7
IL-6/IL-4	7065.3	5,654,200	9,964,700	132,510
IL-6/IL-5	1268.1	191.7	3832.6	26.9
IL-6/IL-9	18.5	7.4	26.6	6.7
IL-6/IL-10	25.4	17.3	219	52.2
IL-12/IL-4	0.00007	0.1	300	270
IL-12 /IL-5	0.00001	0.000003	0.1	0.05
IL-12/IL-9	0.0000002	0.0000001	0.0008	0.01
IL-12/IL-10	0.0000003	0.0000003	0.007	0.1
IFN-α/IL-4	0.7	0.1	0.1	0.01
IFN-α/IL-5	0.13	0.000003	0.00004	0.000002
IFN-α/IL-9	0.0019	0.0000001	0.0000003	0.0000005
IFN-α/IL-10	0.003	0.0000003	0.000002	0.000004
IFN-γ/IL-4	1196	854,500	451,200	41,740
IFN-γ/IL-5	215	29	173.5	8.5
IFN-γ/IL-9	3.1	1.1	1.2	2.1
IFN-γ/IL-10	4.3	2.6	9.9	16.4

## Data Availability

Upon request to the corresponding author.
